# A case report of adult congenital intestinal malrotation

**DOI:** 10.3389/fgstr.2025.1643128

**Published:** 2025-09-18

**Authors:** Yu Gao, Xiaobiao Song, Qiang Song

**Affiliations:** ^1^ Department of Gastrointestinal Surgery, Baotou Central Hospital, Baotou, China; ^2^ Baotou Clinical Medical College, Inner Mongolia Medical University, Baotou, China

**Keywords:** congenital intestinal malrotation, adult, diagnostic, surgical treatment, case report

## Abstract

Congenital intestinal malrotation is a rare congenital digestive tract anomaly, primarily due to the failure of the intestines to rotate and fix properly within the peritoneal cavity during fetal development. This abnormality can lead to severe complications such as intestinal volvulus, intestinal obstruction, and even intestinal necrosis. Although the condition is more commonly seen in neonates, cases in adults have also been reported, often with delayed diagnosis due to atypical symptoms. In this article, we will discuss the diagnosis and treatment of congenital intestinal malrotation in adults.

## 1 Introduction

Congenital intestinal malrotation represents a developmental anomaly of the intestinal tract, characterized by incomplete or aberrant rotational movement of the midgut around the superior mesenteric artery during embryogenesis. This condition demonstrates a high prevalence in the pediatric population, particularly among infants and young children, while its occurrence in adults is exceptionally rare. The clinical presentation in adults is often non-specific, frequently leading to diagnostic challenges, including missed diagnoses, misdiagnoses, and subsequent therapeutic delays. In severe manifestations, congenital intestinal malrotation can pose significant life-threatening complications. This case report presents the diagnostic approach and therapeutic management of an 18-year-old female patient presenting with intestinal obstruction secondary to congenital intestinal malrotation.

### 1.1 Presentation of case

**Figure 1:**
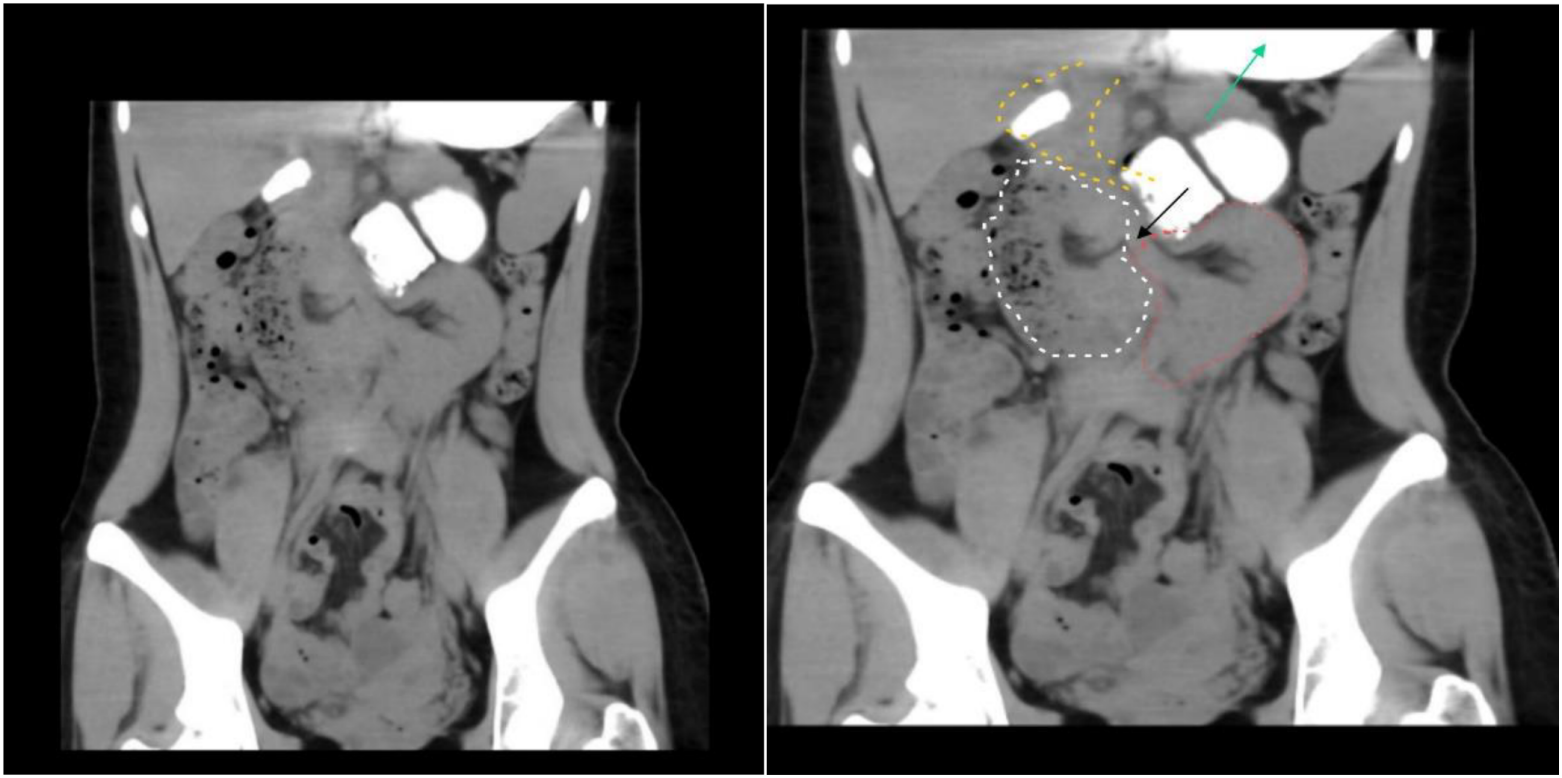
Upon admission, the patient underwent an abdominal CT scan. The stomach (green arrow) and duodenum (the yellow dotted line section) are in normal positions. A narrow ring is formed at the site of the ligament of Treitz (black arrow); the herniated part (the white dotted line section) and the distal intestinal segment (the red dotted line section) present a dumbbell shape.

The patient, an 18-year-old woman, presented to the emergency department with a 24-h history of abdominal distension and discomfort accompanied by nausea and vomiting. The day prior to admission, she developed persistent epigastric distending pain of unknown etiology, associated with multiple episodes of nausea and vomiting. The emesis consisted of gastric contents, and there was no reported cessation of flatus or bowel movements. She initially sought medical attention at an external healthcare facility where conservative management for partial intestinal obstruction was initiated; however, her symptoms showed no significant improvement. Subsequently, she was transferred to our institution for comprehensive diagnostic evaluation and therapeutic intervention. Physical examination revealed a well-developed female adolescent with an acute, distressed facial expression. Cardiopulmonary auscultation demonstrated no abnormalities. Abdominal inspection showed a flat contour without visible gastrointestinal peristaltic waves. Palpation revealed positive tenderness in the epigastric region accompanied by rebound tenderness, but without significant abdominal wall rigidity. Auscultation demonstrated diminished bowel sounds. The patient’s medical history was non-contributory. Ancillary diagnostic investigations, specifically abdominal computed tomography (CT) scan, were performed. The rectal morphology appeared normal. However, there was localized twisting of the jejunal loops within the abdomen, accompanied by dilatation of the adjacent upper and lower intestinal segments, fluid accumulation, and swelling of the intestinal wall. The fat spaces surrounding the lesion exhibited a blurred appearance, with flocculent low-density shadows observed. Additionally, the mesentery was thickened, and multiple enlarged lymph nodes were present in the surrounding area (**Figure 1**).

**Figure 2:**
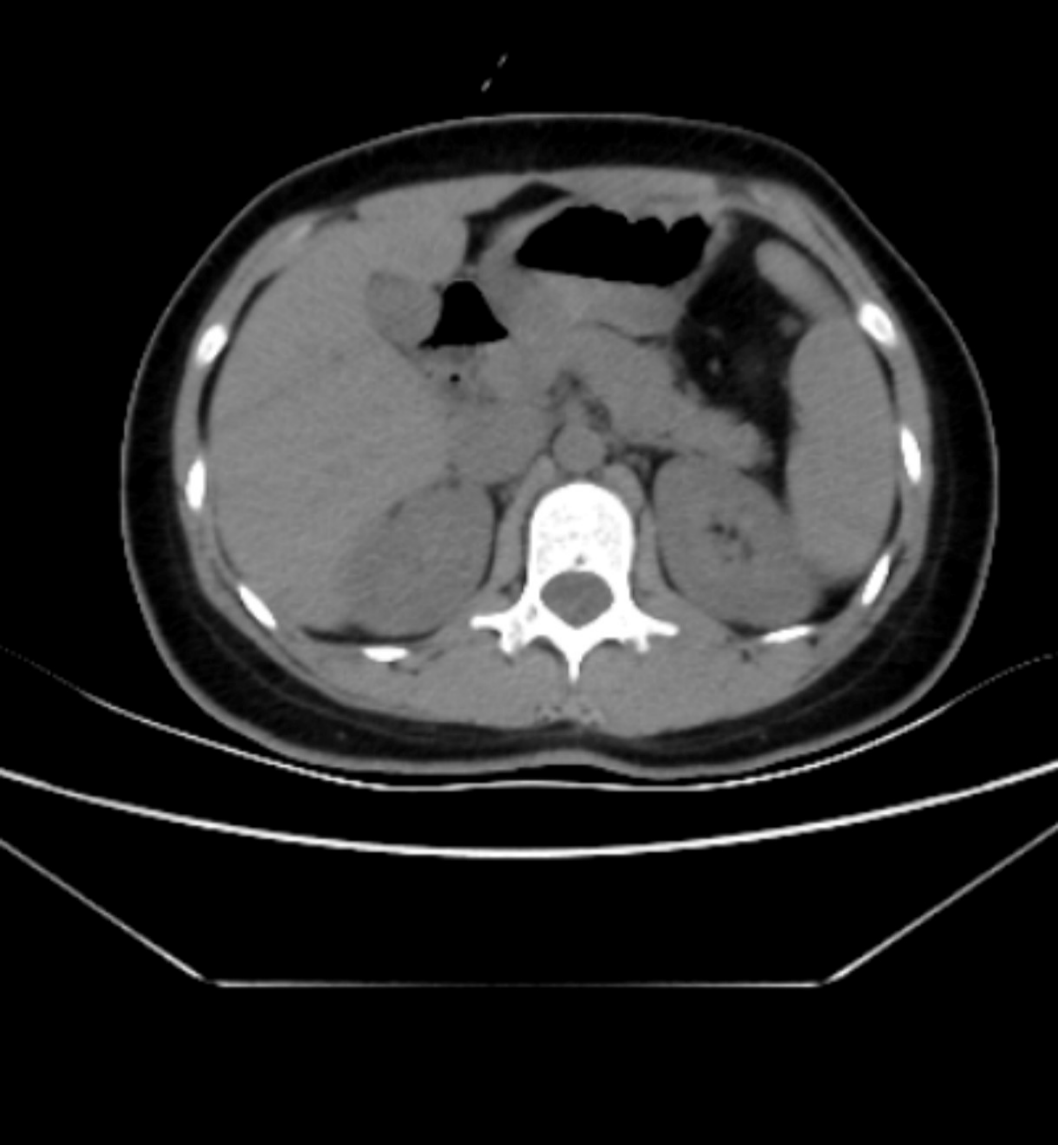
A second abdominal CT scan was performed on the 4th day post-operation. The results revealed that the herniated bowel loops had been successfully reduced, and the obstruction was resolved.

**Figure 3:**
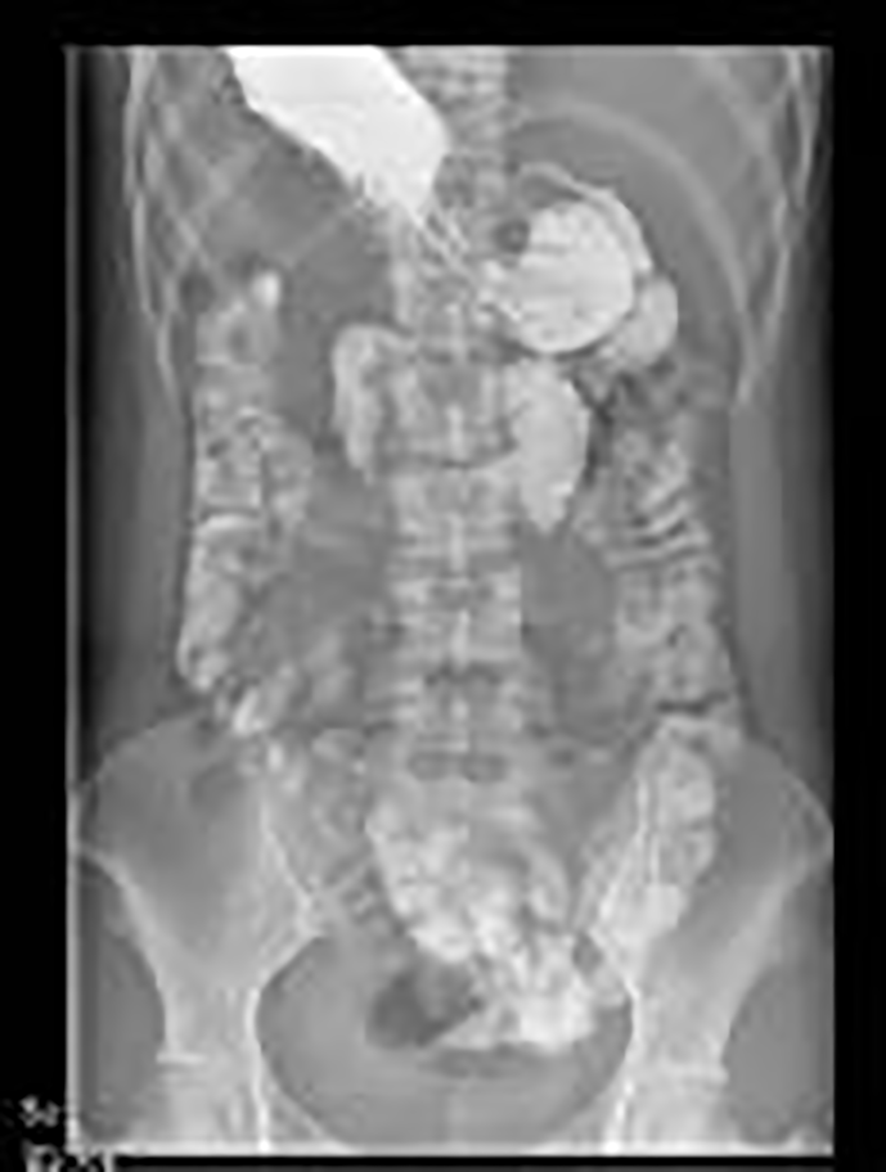
Followed by a gastrointestinal contrast study conducted on the 10th day post-operation, during the follow-up examination conducted 10 days postoperatively, the contrast agent was distinctly visualized traversing the ileocecal junction and subsequently entering the cecal lumen.

After assessing the condition, the patient was urgently admitted to the operating room for laparoscopic exploration. Intraoperative findings revealed a segment of the intestinal tract demonstrating luminal dilation with impaired peristaltic activity, despite maintaining normal serosal coloration. Pathological adhesions were identified between a portion of the greater omentum, the mesenteric attachments of the small bowel, and the right lateral abdominal wall, which were subsequently lysed under direct visualization. A systematic exploration of the entire small intestine was meticulously performed, commencing at the ileocecal junction and progressing cephalad toward the ligament of Treitz. This comprehensive evaluation revealed multiple mesenteric adhesions and fibrotic aggregations; however, these pathological findings did not compromise the vascular integrity or peristaltic function of the intestinal tract. Approximately 110 cm proximal to the ileocecal valve, a significant adhesion was identified between the small bowel and its mesenteric attachments with the transverse mesocolon in the epigastric region, resulting in the formation of a pathological cavity. The mesenteric root at the anatomical ligament of Treitz exhibited an abnormal caudal extension of approximately 10 cm, creating a “pseudo-ligament of Treitz” configuration. A 30-cm segment of proximal jejunum was found herniated into this pathological cavity. The herniated intestinal segment and its associated mesentery demonstrated significant edema, characterized by proximal luminal dilation, erythematous, and thickened intestinal walls, while maintaining adequate vascular perfusion. During the surgical procedure, congenital intestinal malrotation was considered in the differential diagnosis. The adhesiolysis between the intestinal loops and mesenteric attachments was performed with meticulous precision to minimize iatrogenic injury. On the 7th day post-surgery, the patient’s abdominal distension was significantly alleviated, and normal bowel movements were observed **Figure 2**. Following the assessment, a clear liquid diet, including options such as water and broth, was introduced. No complications were observed throughout the entire hospitalization period. The patient’s appetite and digestive function gradually improved, with stable physiological indicators and no signs of infection or adverse reactions; however, the patient had not yet fully recovered (**Figure 3**). By the 13th day post-surgery, the patient had fully recovered, regaining complete mobility and strength, and was discharged after receiving comprehensive postoperative care instructions and a follow-up appointment.

## 2 Discussion

Congenital intestinal malrotation represents a developmental anomaly of the intestinal anatomy, primarily resulting from aberrant or incomplete rotational movement of the midgut around the axis of the superior mesenteric artery during embryogenesis (1). During the fourth gestational week, the primitive gut, comprising the foregut, midgut, and hindgut, extends as an elongated tubular structure from the mesentery. The midgut, centered around the superior mesenteric artery (SMA), initially herniates into the umbilical cord. This segment of the primitive gut undergoes a critical rotational process. By the 10th gestational week, the midgut completes its counterclockwise rotation and subsequent re-entry into the abdominal cavity. Following this process, the duodenum, ascending colon, and descending colon undergo retroperitoneal fixation, establishing the definitive anatomical configuration. The normal anatomical position is characterized by the duodenojejunal flexure situated in the left upper quadrant, superior to the SMA, while the cecum is positioned anterior to the artery and fixed in the right lower quadrant (2–4). Disruption of this developmental sequence, leading to abnormal intestinal rotation and fixation, may predispose to pathological positional alterations of specific intestinal segments, consequently increasing the risk of intestinal volvulus, obstruction, or ischemic complications (2, 5).

### 2.1 Clinical manifestations and symptoms

Congenital intestinal malrotation demonstrates a distinct age-related prevalence pattern, with a significantly higher incidence in pediatric populations compared to adults (6, 7). The clinical presentation of this condition exhibits age-dependent variability, influenced by the degree of intestinal torsion and the development of associated complications. In the neonatal and infantile population, the predominant clinical manifestations include projectile vomiting, abdominal distension, feeding intolerance, and alternating patterns of constipation and diarrhea. In severe presentations, clinical signs may progress to include hemodynamic instability and manifestations of intestinal ischemia or necrosis. In the pediatric population beyond infancy, affected children typically present with chronic abdominal pain, postprandial abdominal distension, recurrent emesis, and persistent constipation, with symptom exacerbation following meal consumption. In adult patients, the clinical presentation is often characterized by chronic, intermittent abdominal discomfort and dyspeptic symptoms, with acute presentations being relatively uncommon in this age group (1, 3, 8, 9).

### 2.2 Diagnostic method

Early and accurate diagnosis is crucial for the optimal management of congenital intestinal malrotation. This diagnostic process can be effectively achieved through the utilization of various imaging modalities, including abdominal computed tomography (CT), radiographic examinations, and ultrasonography. Among these diagnostic tools, abdominal CT has been demonstrated to possess the highest diagnostic sensitivity and specificity. In adult patients presenting with intestinal malrotation, multi-slice spiral CT scanning has proven particularly valuable in delineating the underlying etiology and characterizing the fundamental pathological features of mesenteric edema and intestinal obstruction. This advanced imaging technique has consequently established itself as the diagnostic modality of choice for identifying critical conditions such as mesenteric volvulus and intestinal volvulus (9, 10).

### 2.3 Treatment

Patients with congenital intestinal malrotation often exhibit persistent symptomatology despite conservative therapeutic measures. The development of ischemic intestinal necrosis can precipitate severe clinical complications. Upon confirmation of diagnosis or when congenital intestinal volvulus is strongly suspected, immediate therapeutic interventions should be instituted. These interventions encompass nil per os (NPO) status, gastrointestinal decompression, and fluid resuscitation, coupled with preoperative preparation for surgical management. The surgical intervention of choice typically involves the Ladd procedure, which includes lysis of abnormal intestinal adhesions and anatomical repositioning of the intestinal tract (5, 8). Laparoscopic approaches may be considered as the primary surgical strategy. While this minimally invasive technique generally necessitates extended operative duration, it offers the advantage of reduced postoperative intra-abdominal adhesion formation and shortened hospital convalescence. However, in cases presenting with intestinal obstruction, the presence of intestinal distension and reduced abdominal cavity space may complicate the laparoscopic approach. When laparoscopic procedures cannot be successfully completed, conversion to open surgery should be promptly implemented (11–13). During the surgical intervention, meticulous release of mesenteric and intestinal adhesions is paramount to minimize the risk of postoperative recurrence. In instances where intestinal vascular supply and peristaltic function are compromised and cannot be adequately restored, segmental intestinal resection may be warranted to prevent postoperative intestinal necrosis (9, 14).

## 3 Conclusion

Intestinal malrotation represents a rare clinical entity in adult populations. Furthermore, adult cases frequently exhibit asymptomatic characteristics or present with non-specific symptoms, including chronic abdominal pain, nausea, and vomiting. Due to their insidious nature, many adult cases are often discovered incidentally during imaging studies or surgical procedures, presenting substantial diagnostic and therapeutic challenges due to their non-specific symptomatology and limited clinical exposure among healthcare providers. Secondary intestinal necrosis resulting from volvulus can lead to severe, potentially life-threatening complications. This case report illustrates the clinical course of a patient presenting with intestinal obstruction who demonstrated inadequate response to conservative management, including NPO status, gastrointestinal decompression, anti-inflammatory therapy, and fluid resuscitation. Following emergent surgical intervention, the patient achieved a favorable clinical outcome with complete resolution of abdominal pain, nausea, and vomiting and remained free from postoperative complications. This case emphasizes the critical importance of considering congenital intestinal anomalies in the differential diagnosis of intestinal obstruction with unclear etiology while demonstrating the diagnostic utility of abdominal CT imaging in identifying this condition. Congenital intestinal malrotation typically exhibits poor response to conservative management and carries a significant risk of recurrence. Therefore, when this condition is strongly suspected, it is imperative to promptly communicate the clinical status to the patient’s family and recommend surgical exploration, as the majority of patients can achieve positive outcomes through timely surgical intervention.

## Data Availability

The original contributions presented in the study are included in the article/supplementary material. Further inquiries can be directed to the corresponding author.
